# Draft Genome Sequence of *Flavobacterium* sp. Strain SLB02, Isolated from the Diseased Sponge *Lubomirskia baicalensis*

**DOI:** 10.1128/MRA.00530-20

**Published:** 2020-06-25

**Authors:** Ivan S. Petrushin, Sergei I. Belikov, Lubov I. Chernogor

**Affiliations:** a Limnological Institute, Siberian Branch of the Russian Academy of Sciences, Irkutsk, Russia; b Irkutsk State University, Irkutsk, Russia; Georgia Institute of Technology

## Abstract

There are significant changes in the consortium of microorganisms of freshwater Baikal sponges during their mass death, which began in 2011. The alleged cause of disease is a significant increase in the number of opportunistic microorganisms. Here, we report the draft genome sequence of *Flavobacterium* sp. strain SLB02.

## ANNOUNCEMENT

The first signs of disease of freshwater sponges were discovered in 2011 (1, 2). Sponge disease is accompanied by significant changes in the sponge microbiome. We found that the observed imbalance is caused by several different conditionally pathogenic microorganisms, including members of the families *Chitinophagaceae*, *Oxalobacteraceae*, and *Flavobacteriaceae*. They increase their negative effects by acting together, which leads to the death of photosynthetic microalgae and sponges. The draft genome of one of these conditionally pathogenic bacteria of the family *Oxalobacteraceae*, *Janthinobacterium* sp. strain SLB01, has been published already (3).

In this study, a yellow-pigmented *Flavobacterium* sp. strain was isolated on R2A agar (Merck, Germany) from a sample of diseased sponge (Lubomirskia baicalensis) collected in the littoral zone of Lake Baikal, which is located in central Siberia, Russia. This strain presented as yellow-pigmented colonies on LB agar that had been incubated at 22°C for 48 h. DNA was extracted from the bacterial strain obtained after bead beating using the TRIzol LS reagent (Invitrogen, USA) according to the manufacturer’s protocol.

The sequence library was generated from DNA using an Illumina Nextera XT DNA sample preparation kit. The universal bacterial primers 518F and 1064R (2) were used to amplify the V4 to V6 hypervariable region of the bacterial 16S rRNA gene. Strain SLB02 showed the highest 16S rRNA gene phylogenetic affiliation with species of the phylum *Bacteroidetes* belonging to the family *Flavobacteriaceae.* Whole-genome sequencing was performed using the Illumina MiSeq platform with paired-end chemistry (2 × 250 bp). A total of 17,921,744 paired-end reads were obtained, giving a coverage depth of 470×.

A draft assembly was built using SPAdes v. 3.11.0 (4) with default settings, raw read error correction, and filtering with the built-in Bayes Hammer module (quality threshold, 98%). This draft assembly contained 311 contigs with an *N*_50_ value of 542,632 bp; the largest contig was 940,235 bp.

The resulting contigs were combined into the whole chromosome with Ragout v. 2.3 (https://github.com/fenderglass/Ragout) with default settings (5), using the *Flavobacterium* sp. strain KBS0721 chromosome (GenBank accession no. CP042170) and the Flavobacterium piscis strain CCUG 60099 whole-genome sequence (GenBank accession no. MUHC01000000) as the reference genomes. The final assembly contained one chromosome, with a total genome size of 6,363,829 bp and a GC content of 35.50%.

Genome completeness analysis with BUSCO v. 3.1.0, using the bacteroidetes_odb9 data set with 443 benchmarking universal single-copy orthologs (BUSCOs) and default settings (6), showed results of 96.2% complete BUSCOs, 1.1% fragmented BUSCOs, and 2.7% missing BUSCOs. The genome was then annotated using the NCBI Prokaryotic Genome Annotation Pipeline (PGAP). This assembly contained 4,964 genes, including 4,901 protein-coding sequences, 56 tRNAs, 3 noncoding RNAs, 4 rRNAs, and 73 pseudogenes, as identified by the PGAP.

A search for biosynthetic gene clusters (BGCs) encoding secondary metabolites, using the Web version of antiSMASH v. 5.0 with default settings, predicted the following types of BGCs: type III polyketide synthase, flexirubin, β-lactone-containing protease inhibitor, and bacteriocin BGC (type II lantipeptide synthetase LanM and a nitrile hydratase leader peptide family natural product precursor).

The yellow color is likely due to the presence of carotenoid and flexirubin BGCs. Degradation enzymes present included 44 glycosyl transferases, 4 α-amylases, and metallopeptidases (M1, M13, M15, M28, and M56 families). Cytochrome *cbb*_3_ complex and its oxidase, which are required for successful growth in low-oxygen environments, were found as well.

The availability of the *Flavobacterium* sp. strain SLB02 genome will allow genome-wide comparisons of virulence factors and help in elucidating the underlying genetic requirements for virulence.

### Data availability.

This whole-genome shotgun sequence has been deposited in DDBJ/ENA/GenBank under the accession no. CP045928. Raw reads are available via BioProject no. PRJNA588149 or SRA no. SRR10416295. The version described in this paper is the first version.
